# Genome-to-phenome research in rats: progress and perspectives

**DOI:** 10.7150/ijbs.51628

**Published:** 2021-01-01

**Authors:** Amy L. Zinski, Shane Carrion, Jennifer J. Michal, Maria A. Gartstein, Raymond M. Quock, Jon F. Davis, Zhihua Jiang

**Affiliations:** 1 Department of Animal Sciences, Washington State University, Pullman, WA 99164-7620.; 2 Department of Psychology, Washington State University, Pullman, WA 99164-4820.; 3 Department of Integrative Physiology and Neuroscience, Washington State University, Pullman, WA 99164-7620.

**Keywords:** Rat, Genome sequencing, Genetic variation, Phenome collection, Alternative transcriptomes, Genome-to-phenome

## Abstract

Because of their relatively short lifespan (<4 years), rats have become the second most used model organism to study health and diseases in humans who may live for up to 120 years. First-, second- and third-generation sequencing technologies and platforms have produced increasingly greater sequencing depth and accurate reads, leading to significant advancements in the rat genome assembly during the last 20 years. In fact, whole genome sequencing (WGS) of 47 strains have been completed. This has led to the discovery of genome variants in rats, which have been widely used to detect quantitative trait loci underlying complex phenotypes based on gene, haplotype, and sweep association analyses. DNA variants can also reveal strain, chromosome and gene functional evolutions. In parallel, phenome programs have advanced significantly in rats during the last 15 years and more than 10 databases host genome and/or phenome information. In order to discover the bridges between genome and phenome, systems genetics and integrative genomics approaches have been developed. On the other hand, multiple level information transfers from genome to phenome are executed by differential usage of alternative transcriptional start (ATS) and polyadenylation (APA) sites per gene. We used our own experiments to demonstrate how alternative transcriptome analysis can lead to enrichment of phenome-related causal pathways in rats. Development of advanced genome-to-phenome assays will certainly enhance rats as models for human biomedical research.

## Introduction

The year of the rat started January 25^th^ 2020, and it will end February 11^th^ 2021, based on the lunar calendar. This means Earth recently entered a new repeating 12-year cycle according to the systems of the moon. Nobody knows for certain why the first Chinese emperor assigned the rat as the first sign in the Chinese zodiac, but evidence clearly shows that the species originated in central Asia, including northern China or southwestern China 1. Some of the oldest rat remains can still be found in the border area between Sichuan and Guizhou provinces of China 2. Despite this evidence, the species was mistakenly named* Rattus norvegicus*, as it was thought to have originated in Norway 3. It was, however, the European rat that became the first laboratory animal domesticated for scientific research, the second most used model organism for biomedical purposes and the third mammalian species to have its whole genome sequenced and assembled 3-4.

Rats have been widely used to understand many biological questions in physical, physiological, pathological and psychological sciences for over 150 years 1,3-4. Generally speaking, the animals are used to model many conditions and diseases in humans and mammals, including but not limited to adjuvant-induced arthritis, aging, allergic respiratory disease, behavior, carcinogenesis, carcinogenicity, cardiovascular defects, drug addiction, estrogen-induced pituitary growth, experimental allergic encephalomyelitis, heart failure, hypertension, immunology, induced rheumatoid arthritis, leukemia, longevity, memory, metabolism, motor skills, neoplasia, nephrology, nephropathy, renal degeneration, neurobiology, neurology, nutrition, obesity, insulin resistance, oncology, osteoarthritis, reproduction, reproductive senescence, salt-sensitive hypertension, stroke, teratology, toxicology, transplantation, transplant immunology, vision, hearing, and ophthalmology 5-10. Rats have a relatively short lifespan (usually less than 4 years), making them an ideal model organism to study the many physiological events that humans experience during lifetimes that span up to 120 years.

Traditional approaches, such as divergent selection, crossbreeding and inbreeding as well as modern approaches, including mutagenesis and transgenesis have led to the development of more than 3,000 rat strains worldwide 9. Although a first-generation sequencing method 11 remains in use, next and third generation sequencing technologies have revolutionized genomics and, thus, rat genome analysis 12-13. In parallel, the rat phenome program started more than 15 years ago 14-18. In recent years, transcriptomics, metabolomics and lipidomics have been assimilated into systems biology and other integrated areas, thus advancing our understanding of the complexity of genetic impacts on phenomes 19-25. Certainly, our understanding and appreciation of the complexity behind genetic impacts on phenomes has grown. Here, we review the history, milestones and resources about genome and phenome sciences in rats and discuss approaches, strategies and perspectives in development of genome-to-phenome programs to further promote the rat model in biomedical research.

## Genome Sequencing in Rats

### Strain selection

As shown in Table S1, genome sequencing has been completed on 47 rat strains thus far. The Brown Norway (BN) rat, substrain BN/SsNHsd, was the first strain sequenced based on its broad distribution, popular use and wide availability as well as parenting many inbred strains. Therefore, the BN/SsNHsd rat strain represents the reference genome for the species 3. Interest in genome sequencing and/or re-sequencing of rats then shifted to so-called “founder” strains. For instance, the spontaneously hypertensive rat, SHR/Ola and the Brown Norway derived rat with polydactyly-luxate syndrome, BN-*Lx* served as the founders to form the rat HXB/BXH recombinant inbred (RI) panel 26. The SHR/OlaIpcv strain (an inbred strain derived from SHR/Ola) and the BN-*Lx* rat were sequenced by two teams 27-28 and the eight founders of the outbred heterogeneous stock (HS) rat- ACI/N, BN/SsN, BUF/N, F334/N, M520/N, MR/N, WKY/N and WN/N- have been sequenced twice, but analyzed three times 26, 29-30.

The ultimate goal of rat genome sequencing is to model genetic complexity of complex disease phenotypes relevant to humans. Therefore, 29 rat stains, including ACI/EurMcwi, BBDP/Wor, F344/NCrl, FHH/EurMcwi, FHL/EurMcwi, GK/Ox, LE/Stm, LEW/Crl, LEW/NCrl, LH/MavRrrc, LL/MavRrrc, LN/MavRrrc, MHS/Gib, MNS/Gib, SBH/Ygl, SBN/Ygl, SHR/NHsd, SHRSP/Gla, SR/Jr, SS/Jr, SS/JrHsdMcwi, WAG/Rij, WKY/Gla, WKY/NCrl, WKY/NHsd, BN-*Lx*/CubPrin, SHR/NCrlPrin, SHR/OlaIpcvPrin and SUO_F344 were sequenced to screen the artificial selective sweeps associated with cardiovascular and metabolic phenotypes 26,31. In addition, WGS was also performed on 7 strains: DA/BklArbNsi, F344/NHsd, DA/hanKini, DA/OlaHsd, E3/han, PVG/1AV1.Kini and HSRA to understand the differences in susceptibilities to arthritis, autoimmunity, inflammation, cancer and congenital abnormalities of the kidney and urogenital tracts 32-34.

### Sequencing and processing

**Figure 1:**
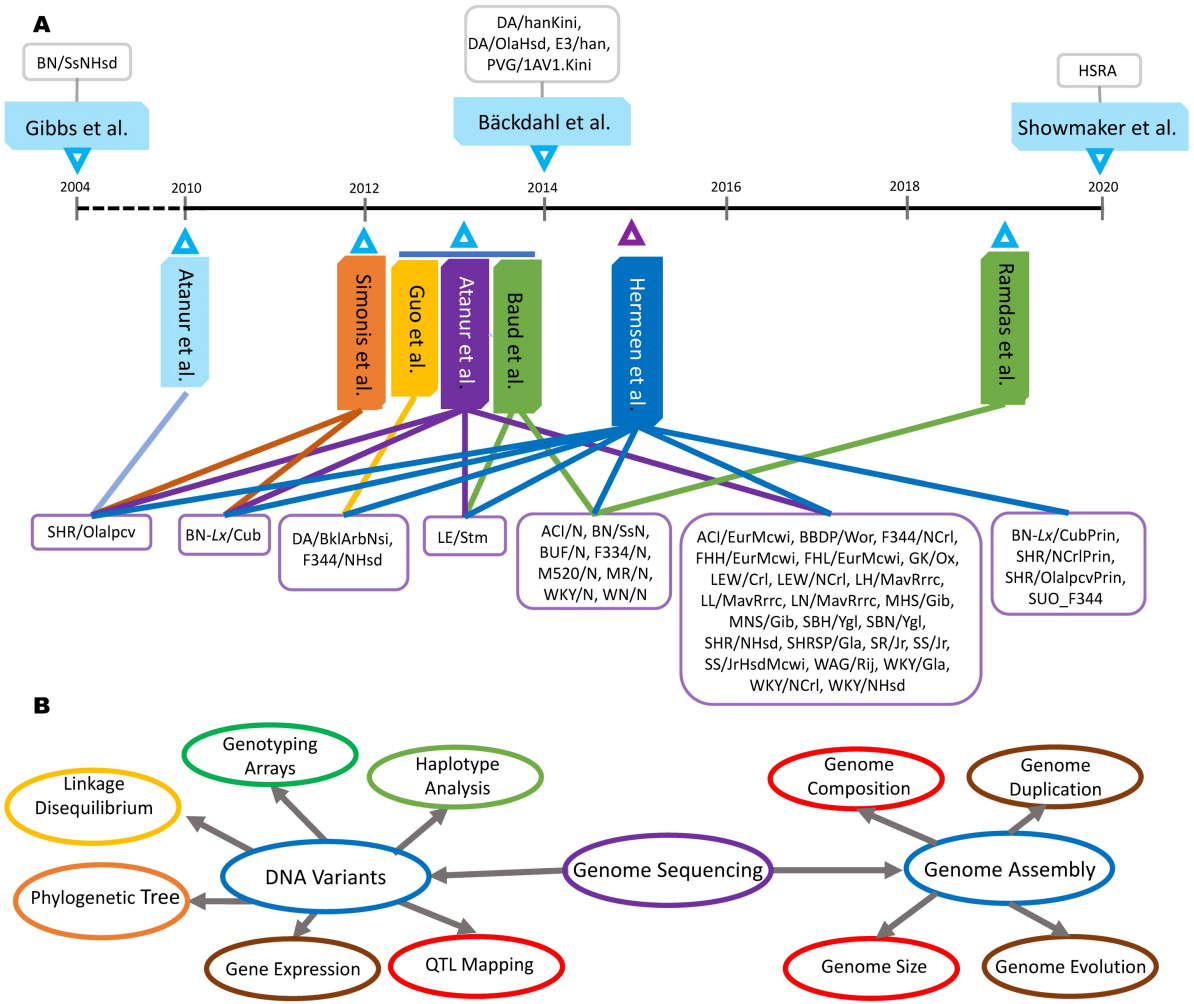
** Genome sequencing and applications in rats. A**: Forty-seven rat strains were used to pursue whole genome sequencing or re-sequencing by ten teams. **B**: Genome sequences have been used to complete genome assemblies and detect the DNA variants for various applications.

As illustrated in **Figure 1**, 45 of 47 rat strains had whole genomes sequenced from 2010 - 2015, making this the most prolific period in rat genome sequencing history. Among these 47 rat strains, only the BN/SsNHsd rats were sequenced using the Sanger method 11, while the remaining 46 rat strains were sequenced on either SOLiD 28-29, 33 or Illumina platforms 26-27, 30-32, 34. In terms of sequencing depth, the lowest genome coverage was 7× for the BN/SsNHsd rat, followed by ~10× for BBDP/Wor and ~12× for WKY/NHsd. Most of the rat strains were sequenced with 18-33× coverage. The most recently sequenced strain, HSRA, had more than 47x genome coverage (Table S1).

The Atlas software package was specifically designed to assemble the initial sequences of the rat genome 35. It used DNA sequence reads derived from both BACs and whole genome shotgun libraries and gradually improved the sequence maps using BAC fingerprinting maps, physical and linkage maps, and comparative maps with other species. The initial assembly of rat genome was named Rnor3.1 3. Mapping next generation sequencing (NGS) short reads to the reference genome has been primarily completed using MAQ (mapping and assembly with quality 36), SOAP (short oligonucleotide alignment program 37) and BWA (Burrows-Wheeler Alignment Tool 38). Additionally, SAMtools (Sequence Alignment/Map format tool 39) and GTAK (Genome Analysis Toolkit 40) have been popular tools to call genome-wide variants in rats (Table S1).

### Improvement and characterization

The first draft genome of the rat was 2.75 Gb in length, 60% of which came from whole genome shotgun sequencing and 40% from BACs 3. In 2013, both DA (Dark Agouti) and F344 (Fischer) rats were sequenced with an average depth of 32× for whole genome assembly 32. The authors created a new strategy combining *de novo* assembly with reference-aided assembly, which generated 2,616,053,766 bp for the former strain and 2,615,410,193 bp for the latter strain. The new genome drafts generated by their approach added ~50 million base pairs of novel sequences to the BN/SsNHsd genome and closed more than 400,000 gaps. Despite this improvement, the early version of the rat genome assembly remained incomplete. After aligning WGS data from 33 rat strains to the Rnor5.0 reference genome, for example, van der Weide *et al* 41 found that the unmapped reads ranged from 2 million to 150 million bp per strain.

Fortunately, 10× long-reads generated by the PacBio sequencer improved the rat genome assembly and led to the release of Rnor6.0 (2,782.07 Mb in length) 42. Overall, the rat genome is 306.27 Mb shorter than the human genome (3,088.3 Mb, GRCh38.p13 release 109), but is 56.51 Mb longer than the mouse genome (2725.52 Mb, GRCm38.p6 release 108) (**Table 1**). These updates further confirm the initial claim released in 2004 that genome size order among these three species is: human (2.9 Gb) > rat (2.75 Gb) > mouse (2.6 Gb) 3. The same order is also true for the large segmental duplications with human (5 - 6%) > rat (3%) > mouse (1 - 2%). In contrast, the order is reversed for the median GC content in these three species: mouse (42.4891%) > rat (42.3423%) > human (40.9%) (**Table 1**).

The nuclear genome has 23, 20 and 21 pairs of chromosomes in humans, mice and rats, respectively (**Table 1**). Chromosome-wide painting of sequences using the orthologous genes as landmarks can reveal the synteny blocks between a pair of species. The BAC-based analysis, for instance, showed that there are 146 synteny blocks between the rat and mouse, while there are 295 blocks between human and rat, and 299 blocks between human and mouse 43. An early study also showed that there are more than 5,000 ultraconserved 100-bp elements with identical sequences among human, rat and mouse 44. After investigation of DNA methylation patterns in blood, brain and sperm samples in all three species, Zhou and co-workers 45 discovered that 88% and 57% of the rat tissue-specific differentially methylated regions have orthologous counterparts in mouse and human, respectively. Based on the gene information released so far, however, we argue that the structural and functional annotations of the rat genome are inferior to both human and mouse annotations (**Table 1**).

### Genome Biology in Rats

#### Genome variation

Of the 47 rat strains with whole genome sequences (Table S1), the BN/SsNHsd strain was considered too pure to produce large amounts of genome variation, even when assembled with over 36 million sequence reads 3. Because the BN/SsNHsd strain has been used to produce the rat reference genome, sequencing of closely related strains, such as BN/SsN, BN-Lx/CubPrin and BN-Lx/Cub revealed no more than 150,000 SNVs (single nucleotide variants), 700,000 INDELs (insertions/deletions) and 15,000 SVs (structural variants) from sequencing data reported from 2012 to 2015. However, other datasets published during the same timeframe revealed that the genomes of more divergent rat strains possess 2,664,124-3,819,860 SNVs, 151,099-1,725,868 INDELs and 2,301-58,877 SVs in reference to the BN/SsNHsd genome. These variations may provide evidence that the current bioinformatics pipelines can call SNVs or SNPs (single nucleotide polymorphisms) more accurately than INDELs and SVs, because re-analysis of the same datasets produced dramatic differences in numbers of the latter two genome variants (Table S1). In addition, laboratories can affect variant identification. Eight rat strains: ACI/N, BN/SsN, BUF/N, F334/N, M520/N, MR/N, WKY/N and WN/N, for example, were sequenced twice and re-analyzed three times. Baud *et al.* 29 and Hermsen *et al.* 26 detected 71,038 and 59,402 SNVs in the BN/SsN rat strain, respectively, compared to 2,364,508 CNVs detected by Ramdas *et al.* 30. For the remaining seven strains, 2,664,124 - 3,213,913 SNVs were reported by the first two laboratories, while 7,107,048 to 7,518,136 SNVs were collected by the third laboratory (Table S1).

Using only a few animals per strain in WGS or re-sequencing experiments may result in limited information about genome variation in rats. In cases such as this, the false-negative rate will be much higher than the false-positive rate. When SOLiD and capillary variant calls were compared, for instance, Baud and colleagues 29 found that the false positive rate was 2.7% for SNVs, 2.2% for INDELs and 16.7% for SVs, but the false negative rate increased to 17.2%, 41.4% and 65% for the same types of variants, respectively. In addition, WGS of only a few rats per strain makes it difficult to detect heterozygous loci so that the ratio between homozygous and heterozygous variants is extremely high. Among 2,964,158 SNVs in DA/BklArbNsi and 2,973,513 in F344/NHsd, 95% (2,816,017 and 2,816,677, respectively) are homozygous SNVs 32. After examining 9,665,340 SNVs across 27 rat strains, Atanur *et al* 31 found that 98.3% of the SNVs were homozygous. Furthermore, the definitions for INDELs and SVs are too simple and arbitrary. Atanur and co-workers 27 defined insertions or deletions ≤ 15 bp as INDELs, but > 50 bp as SVs. It is unclear how the authors handled the INDELs between 15 bp and 50 bp in length. As shown in **Table 2**, almost 99% of SNVs are located in intergenic (including upstream and downstream regions) and intronic regions, while only 1% are expressed SNVs in rats.

DNA variants, specifically SNVs or SNPs, can be placed on high-throughput genotyping platforms, such as Illumina BeadLab station and Affymetrix genotyping arrays. For example, seven 1,536-plex assays with a total of 10,752 SNPs on the Illumina BeadLab station and a 10K (9,691 SNPs) rat Targeted Genotyping panel on an Affymetrix platform were designed by Saar and colleagues 46. A high density Affmetrix genotyping array called RATDIV with a total of 803,485 SNPs was also developed by Baud and colleagues 29. These novel tools enable genotyping of multiple strains and/or individuals to complete phylogenetic trees, genome-wide haplotype structures and genetic linkage maps 29,46. These tools significantly advanced QTL (quantitative trait loci) mapping in rats.

#### QTL mapping

QTL mapping is the most popular approach to detect genetic information flows from genome to phenome 33,46-47. Combining WGS with QTL mapping has become more detailed, accurate, and inclusive, leading to more discoveries of genes and loci related to diseases and other phenotypes of interest 47. Because rats are one of the most studied model organisms, many QTLs have been discovered in the species 31,46. For example, Bäckdahl and colleagues 33 identified specific causal genetic mutations detailing how they disrupted gene function such as non-synonymous mutations, additions or deletions of stop codons, and higher instances of SNVs in splicing sites and UTRs (untranslated regions).

Haplotype structure has also been explored in rat QTL mapping. Baud and colleagues 29 used the haplotype association in both mixed models and resampling methods and thus mapped 355 QTLs linked to 122 phenotypes (an average of 2.9 QTLs per phenotype) by genotyping and phenotyping 1,407 HS rats. Of these QTLs, 22 had effect sizes larger than 15%, making large effect QTLs rare (6%) and small effect size QTLs common (average effect size of 6.5%, median effect size of 5%). Very small effect QTLs were also rare (7.8%), as only 28 had effect sizes less than 2.5%. The average heritable phenotypic variance explained by the QTLs was 42%. In particular, the team observed that 38% of QTLs (131 QTLs) contained one or more candidate genetic variants.

Linking these so-called putative artificial selective sweep (PASS) regions to previously identified QTLs presents another unique approach to discover genetic variants underlying complex phenotypes among founder rat strains 31. Some PASS regions were unique to one rat strain, while others were shared among the strains of a disease model. There were 15,859 PASS regions (20 kb to 2.9 Mb in length) that were positively selected during the derivation of disease model strains, as they contained significantly higher levels of LD (linkage disequilibrium) and non-synonymous SNVs than non-PASS regions of the genome. Furthermore, these PASS regions likely contain genes and gene clusters responsible for the phenotypic differences between rat strains with and without the region(s) and most PASS regions either contained QTLs or were near (less than 10 Mb) QTLs. This is an excellent example of QTL utility because it provides a better description of the differences between strain phenomes from a genomic standpoint and, in some cases, specific candidate genes were identified through the PASS region x QTL comparisons. Thus, by juxtaposing QTLs to PASS regions, which are essentially specialized haplotype blocks, identifying shorter causal genomic regions and genes is more feasible.

In addition to these advanced QTL mapping strategies developed in rats, RI and HS strains have also contributed to quantitative genetic studies. These rat strains have been used to detect QTLs 48-61 for 1) cardiovascular conditions, such as blood pressure, electrocardiographic parameters, basal mean arterial pressure (MAP), delta MAP and delta heart rate, left ventricular hypertrophy and heart size; 2) metabolic complexity, such as alcohol consumption and propensity for alcohol-induced organ damage, glucose oxidation and its incorporation into brown adipose tissue lipids, insulin resistance, dyslipidemia and glucose handling; 3) behavioral traits, such as shuttle box, automated novel cage activity and elevated zero maze, startle motor response and habituation, anxiety and locomotion traits associated with elevated plus maze, and conditioned taste aversion; 4) physiologic and hormonal quantitative traits, such as serum adiponectin and thyroid stimulating hormone activity, dopamine beta-hydroxylase enzyme activity, catecholamine biosynthesis and catecholamine secretion; 5) bone fragility, bone mineral density, bone structure and bone strength; 6) hematology, such as full blood count; 7) immunology, such as FACScan analysis of white blood cells; 8) neuroinflammation, such as myelin oligodendrocyte glycoprotein induced experimental autoimmune encephalomyelitis and TNF alpha in serum; and 9) tissue weight, such as adrenal glands, bone, blood, brain, ears, heart, kidney, liver, spinal cord, spleen, and thymus. In addition, Szpirer 62 recently reviewed the use of rats for knockout investigations of QTLs and gene functions.

#### Broad application of genetic markers

**Figure 2:**
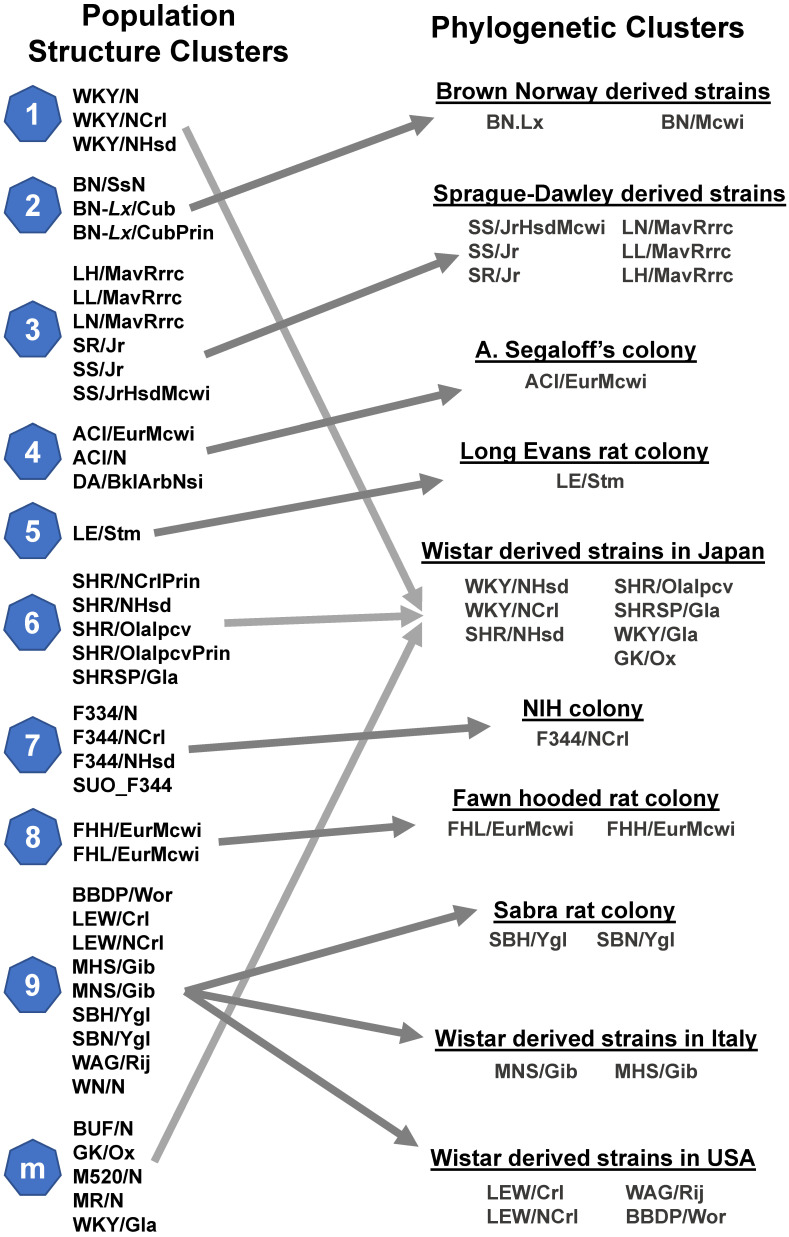
** Evolutionary relationships among rat strains.** Population clusters were constructed through the genetic profiling of 9,183,702 SNVs in a Bayesian model 26. Each cluster was assigned a number, with cluster m denoting (mixed) rat strains that inherited genetics from multiple other clusters. Phylogenetic clusters were constructed using 9.6 million SNVs and the FitchMargoliash method 31.

DNA variants can serve as genetic markers to reveal the origins or evolutionary relationships among rat strains. Hermsen and colleagues 26 delineated 41 rat strains into 9 population clusters and one multiple-origin cluster, while Atanur and colleagues 31 clustered 27 of those strains using phylogenetic analysis. As shown in **Figure 2**, the structure clusters 1, 6 and m (part of the multiple-origin group) contributed to a phylogenetic cluster whose founder was a Wistar strain derived in Japan. The structure cluster 9 was split into three phylogenetic clusters: Sabra rat colony, Wistar-derived rat strains in Italy and Wistar-derived rat strains in the US. The remaining clusters matched between the two studies (**Figure 2**). These results indicate that both analyses were highly correlated, demonstrating that comparing genetic variants between strains is a successful and reliable method of determining the phylogenetic relationships of inbred rat strains.

Haplotype blocks are regions of the chromosome that display low levels of recombination and can therefore be defined by a limited number of alleles or instances of genetic variation. Therefore, haplotype blocks identify regions of the genome with low rates of LD and a shared genetic background (*i.e.* conserved ancestral portions of the genome) between individuals or strains. Usually, detection of stable and detailed haplotype blocks requires a relatively large sample size - several hundreds to thousands of samples yield the most dependable results 63-64. In 2008, Saar and colleagues 46 completed the construction of 837 haplotype blocks (411 kb average block size), covering 12% of the rat genome and including 19% of total SNPs. With this information, the team concluded that the laboratory rat founder population contained more genetic variation, thus causing difficulties in phylogenetically mapping inbred rat strains. Furthermore, 939 inter-chromosomal SNP pairs were classified as being in full LD, which were also related to specific sections of the rat phylogenetic tree. In short, these SNPs and haplotype blocks were able to produce a more detailed history of the genetic divergence between inbred rat strains.

There are several interesting features about genome variants and their effects on gene expression in rats. When genes gain stop codons, lose start and stop codons or change splicing donor or acceptor sites, they are rarely expressed 26. This phenomenon is also confirmed by Simonis and colleagues 28 who found that only 4% of the differentially expressed genes between BN-*Lx* and SHR contain a stop variant. If a gene is fully deleted, causing the structure variation, it would not be expressed. However, gene duplication can result in significant changes in expression. Among seven fully duplicated genes, for example, five were differentially expressed in the liver between BN-*Lx* and SHR rats 28. The variant density in the 5 kb upstream of the transcriptional start site seems to affect gene expression because the differentially expressed genes contain, on average, 8.1 SNVs and 2.0 INDELs in the region in comparison to non- differentially expressed genes that possess 6.6 SNVs and 1.7 INDELs on average (two tailed *t*-test, *P*-value 0.0003 and 0.005 for SNVs and INDELs, respectively) 28.

### Phenome Collection in Rats

#### Rat phenome project initiatives

More than 15 years ago, the genome community initiated the rat phenome projects to further coordinate genome resources and their applications to improve the value of this model organism 14-16. The goals of the rat phenome projects are to systematically characterize various rat strains with standardized quantitative and qualitative parameters, thus promoting laboratory rats as biomedical models to study physiology, phycology, pathology and pharmacology relevant to human health. The phenome datasets collected from diverse rat strains allow development of phenotypic ranking systems that can be easily used to visualize the normal ranges of phenotypes, determine the parameters of disease-based phenotypes, simultaneously compare phenotypic values among many strains and improve experimental designs in terms of reliability and reproducibility 14. Understanding the phenotypic differences between strains will also help researchers identify which strains to select for future QTL analyses 14. Other factors, such as sex and age are included to characterize the phenotypes. No doubt, these phenome projects have and will continue to provide resources and reagents to build links between genomes and phenomes 16.

#### The U.S. rat phenome program

**Figure 3:**
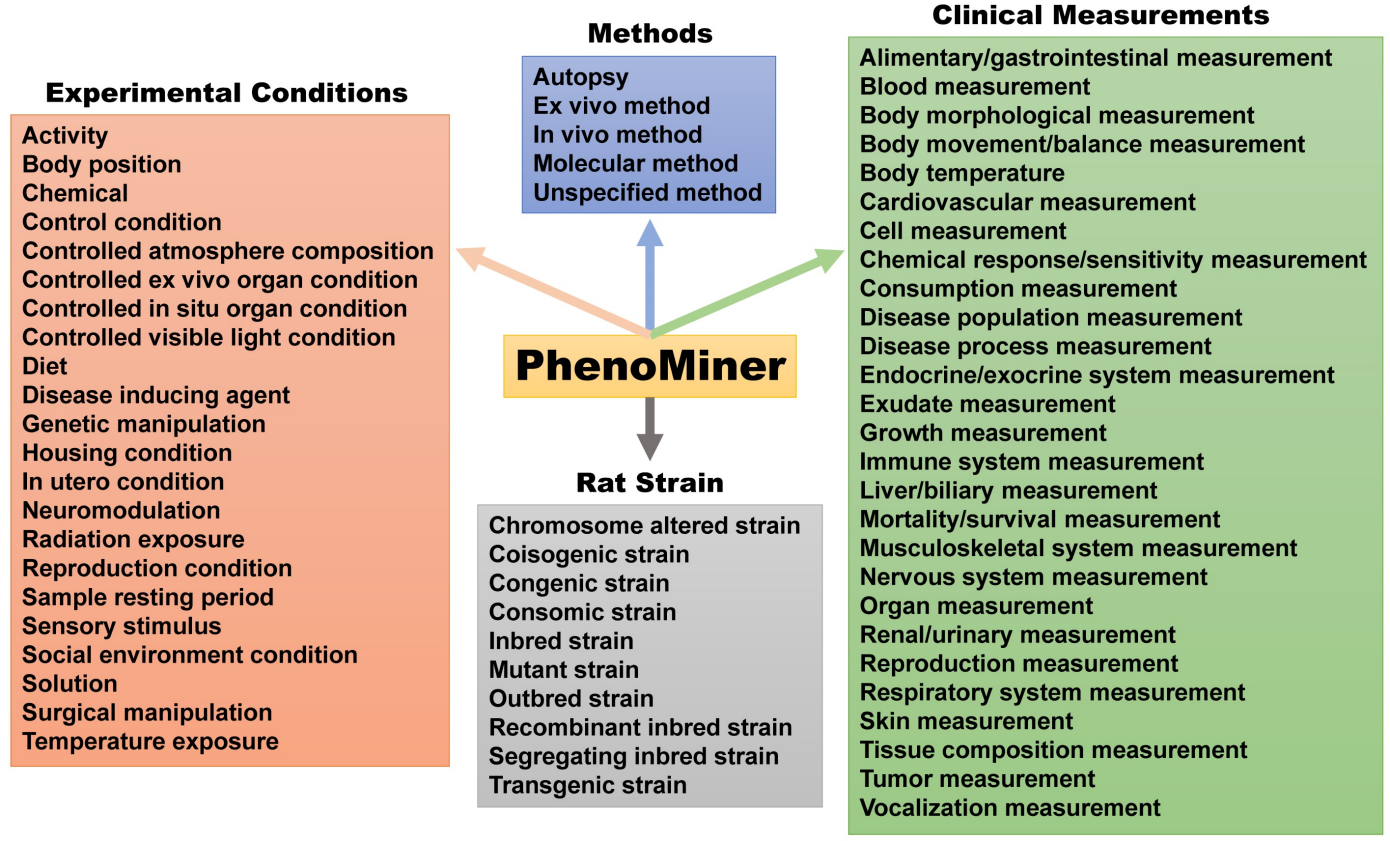
** The PhenoMiner search engine created by the US phenome program.** This tool can search for methods, experimental conditions and clinical measurements collected for each rat strain.

The Medical College of Wisconsin pioneered development of the US rat phenome resources 15. The rat genome database (RGB) is a phenome-related website that harbors information on genetic models, PhenoMiner, expected ranges of phenotypes, PhenoMiner term comparisons, phenotypes of rats and other animal models (https://rgd.mcw.edu/wg/physiology/). For genetic models, the website lists 396 gene-specific rat models, including approximately 50 strains with phenotype information. PhenoMiner is a search engine for retrieving data on rat strains, experimental conditions, clinical measurements and measurement methods (**Figure 3**). Expected ranges of quantitative phenotypes are categorized into blood homeostasis, body mass and body temperature, circulatory system morphology, circulatory system physiology, connective tissue morphology, gland morphology, grooming behavior, hemolymphoid system morphology, hepatobiliary system morphology, immune system morphology, kinesthetic behavior, molecule homeostasis, nervous system morphology, reproductive system morphology, respiratory system morphology, respiratory system physiology and urinary system morphology traits, respectively (Table S2). With many independent phenotyping studies and projects, phenotypical data for the rat became plentiful in the early 2000s, but most of it was not standardized 65. In an effort to solve this issue and catalog data in a way that allowed for cross-strain comparisons, Zhao and colleagues 65 created the Expected Ranges tool available at the RGD website. In addition to presenting the normal ranges of the phenotypes for strains to researchers, the Expected Ranges tool also predicts the ranges of these phenotypes given specific experimental conditions. In this way, researchers have access to phenotypical estimates tailored for not only the rat strains, but also laboratory conditions to determine which strains are the most appropriate for their research protocols.

#### The Japanese rat phenome program

The National Bio Resource Project for the Rat (NBRP-Rat), a subdivision of the National Bio Resource Project of Japan, is a repository and distributer of over 250 inbred rat strains (http://www.anim.med.kyoto-u.ac.jp/nbr) 14. Established in 2002, the project has collected over 109 measures of phenotypic data to fit the following seven categories: anatomy, behavioral studies, biochemical blood tests, blood pressure, hematology, neurobehavior, and urology (Table S3). The NBRP-Rat also houses 370 SNPs collected from over 50 rat strains to further integrate genotypic and phenotypic data. Their goal is to screen 200 rat strains for SNPs as well as phenotypic markers to increase the value of the rat as a model organism and identify valuable phenotypic differences between inbred strains. In this way, normal and abnormal phenotypes can be identified and distinguished between inbred rat strains. For example, in 2005, the data mined from NBRP-Rat showed that SHR strains have the heaviest heart weights, while the hypertensive ZI rat strain have a normal heart weight and size 14, demonstrating that hypertensive ZI rats develop high blood pressure differently than SHR rats.

#### Phenome collection on the BN rat

Kwitek and colleagues 16 were the first to complete a large-scale phenotyping project on the BN rat. They compared 281 phenotypic trait measurements (focusing on renal, pulmonary, and cardiovascular traits) collected from the BN rat to 10 other inbred and outbred strains (CDF, CD®IGS, FHH, GH, LE, LEW, SD, SHR, SS, and WKY) and found that no one strain serves as a representative control. Instead, they found that the normal ranges of phenotypes are relative and vary between strains. The disparity in phenotypes between genders also varied between strains. In response, Kwitek and colleagues 16 combined the collected data from all strains into a 'population' dataset and determined normal ranges of the phenotypes that all strains can be compared against. Their data can be found on the Rat Genome Database (RGD) website (https://rgd.mcw.edu/wg/phenotype-data13/). Of the traits measured, 39% were significantly different in the BN rat as opposed to the 'population' mean and 46% of the respiratory and lung traits were determined to be significantly different, supporting the notion that the BN rat is sensitive to inflammatory lung diseases, such as asthma, and should be utilized in respiratory research. In comparison, only 15% of renal-related phenotypes were significantly different from the population mean, suggesting BN rats could also serve as a control strain in renal function studies, further demonstrating that selection of a rat strain for research is dependent on the focus of the study.

#### Phenome collection for QTL mapping

Baud and colleagues 29 collected a total of 160 phenotypes for their work. The phenotypes were grouped into anxiety, arterial elastic lamina ruptures, body weight, bone morphology, cardiovascular function, coat color, glucose tolerance, hematology, immunology, induced neuroinflammation, renal agenesis, serum biochemistry and wound healing (Table S4). Depending on the trait, various covariates were included in the QTL analysis. A genome-wide association study (GWAS) was performed using the mixed models and resampling methods. The number of animals varied from 185 to 1407 rats, including both sexes. In combination with WGS, the team revealed 355 QTLs for 122 complex phenotypes and identified 35 causal genes for 31 phenotypes. As such, this work presents one of the most successful QTL mappings in rats.

#### Phenome collection for drug abuse and addiction research

Drug abuse and addiction significantly burden society in terms of health care costs, loss of productivity, crime and mortality 66-67. Thus, research on how to best prevent and treat these psychological disorders is valuable. The rat has been widely used to not only discover phenotypes associated with various substance use disorders (SUDs) but has also been used to understand the molecular mechanisms and genes associated with SUDs (Table S5). Recently, the validity of rat and other animals as models for SUDs have been has been questioned; however, behavioral and physiological data suggests that rats are not only appropriate, but quite excellent models of SUDs in humans 66-67. Rats are capable of modeling many psychological and physiological aspects of SUDs including drug seeking behaviors, the reinforcing effects of various drugs, factors affecting susceptibility to drug addiction (environmental and genetic), withdrawal symptoms, causal molecular pathways of addiction, epigenetic changes in the brain and other tissues in response to drugs, and the feasibility of novel treatment strategies 66-67 (Table S5). Amongst these factors, differences in gender, age, and drug type have all been successfully identified 66,68-70. For instance, while all drugs impact the dopamine signaling of dopaminergic neurons in the brain to cause addiction - these neurons comprise the regions of the brain responsible for reward seeking behaviors and motivation (the reward system) - some impact dopamine signaling directly, while others affect the system indirectly 67.

#### Public databases for genome and phenome resources in rats

**Figure 4:**
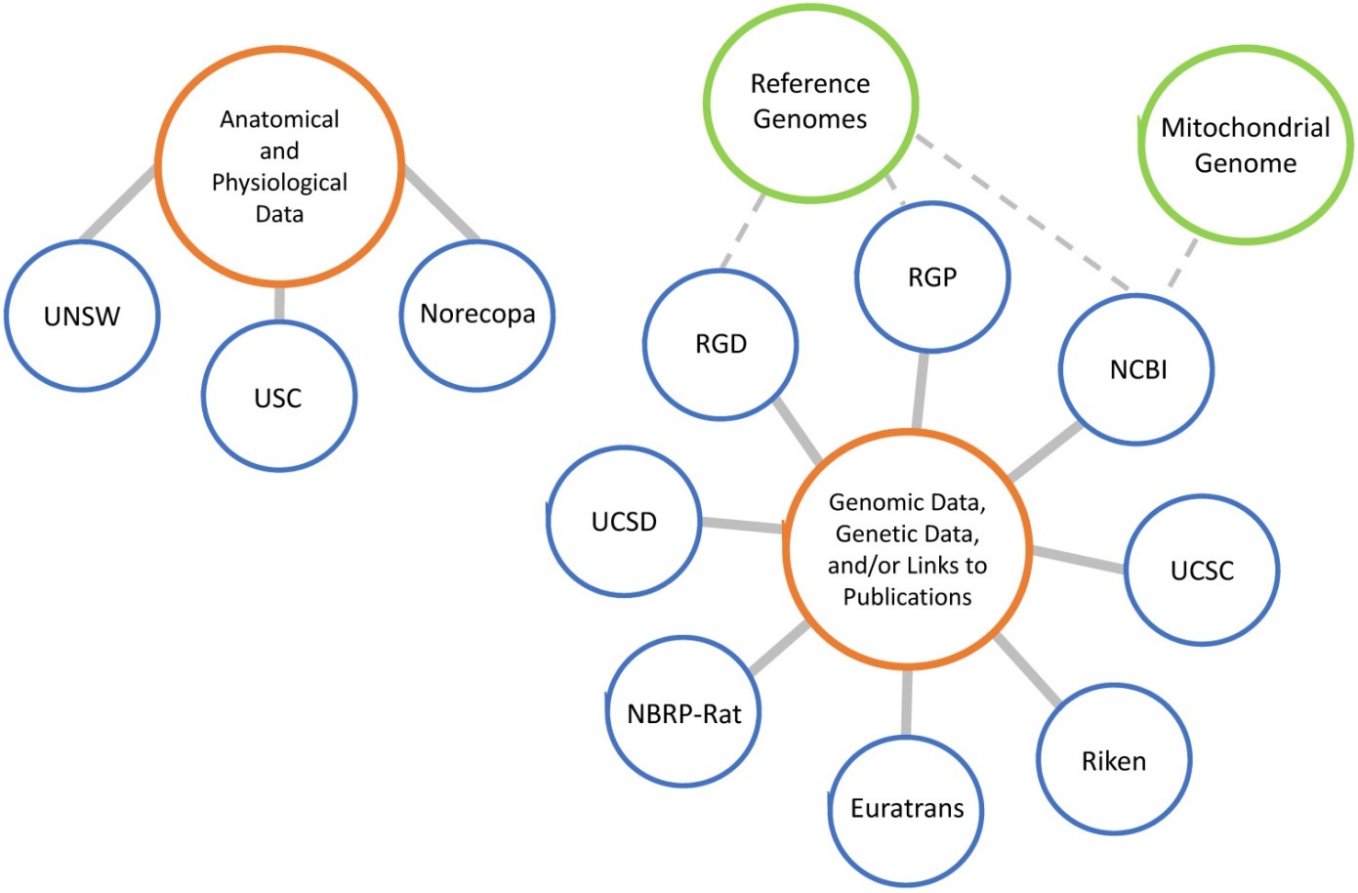
Worldwide rat genome and phenome databases.

Many databases pertaining to the rat are available; however, each one is unique in that not all of them present the same information and resources. In general, databases store anatomical or physiological data, genetic and genomic data, catalog rat strains, or catalog laboratories that develop and maintain rat strains (**Figure 4**). Beyond this, each database has its own niche, with specific data that may be more valuable to some research interests than others. The US has established at least six genome, phenome and/or GWAS databases, including 1) Rat Genome Database at the Medical College of Wisconsin (http://rgd.mcw.edu/), 2) Rat Genome Resources at National Center for Biotechnology Information (http://www.ncbi.nlm.nih.gov/genome/guide/rat/index.html), 3) Rat Genome Project at the Baylor College of Medicine (http://www.hgsc.bcm.edu/other-mammals/rat-genome-project), 4) Rat Genome Browser at UC Santa Cruz (https://genome.ucsc.edu/cgi-bin/hgGateway), 5) Rat Atlas at University of Southern California (https://loni.usc.edu/research/atlases) and 6) Genes and Addiction - NIDA Center for GWAS in Outbred Rats at UC San Diego (https://ratgenes.org/). Other databases include 1) Ensemble at the European Molecular Biology Laboratory's European Bioinformatics Institute (http://uswest.ensembl.org/Rattus_norvegicus/Info/Index), 2) European large-scale functional genomics in the rat for translational research (http://www.euratrans.eu/), 3) National BioResource Project for the Rat in Japan (http://www.anim.med.kyoto-u.ac.jp/nbr/), 4) Riken Bioresource Center (https://dna.brc.riken.jp/en/gene_expressionen/hoststrainen), 5) University of New South Wales (UNSW) Embryology (http://embryology.med.unsw.edu.au/embryology/index.php/Rat_Development), and 6) Norecopa (https://norecopa.no/films-and-slide-shows).

### Genomes to Phenomes: What are the Bridges?

#### Progress in bridging the gaps

**Figure 5:**
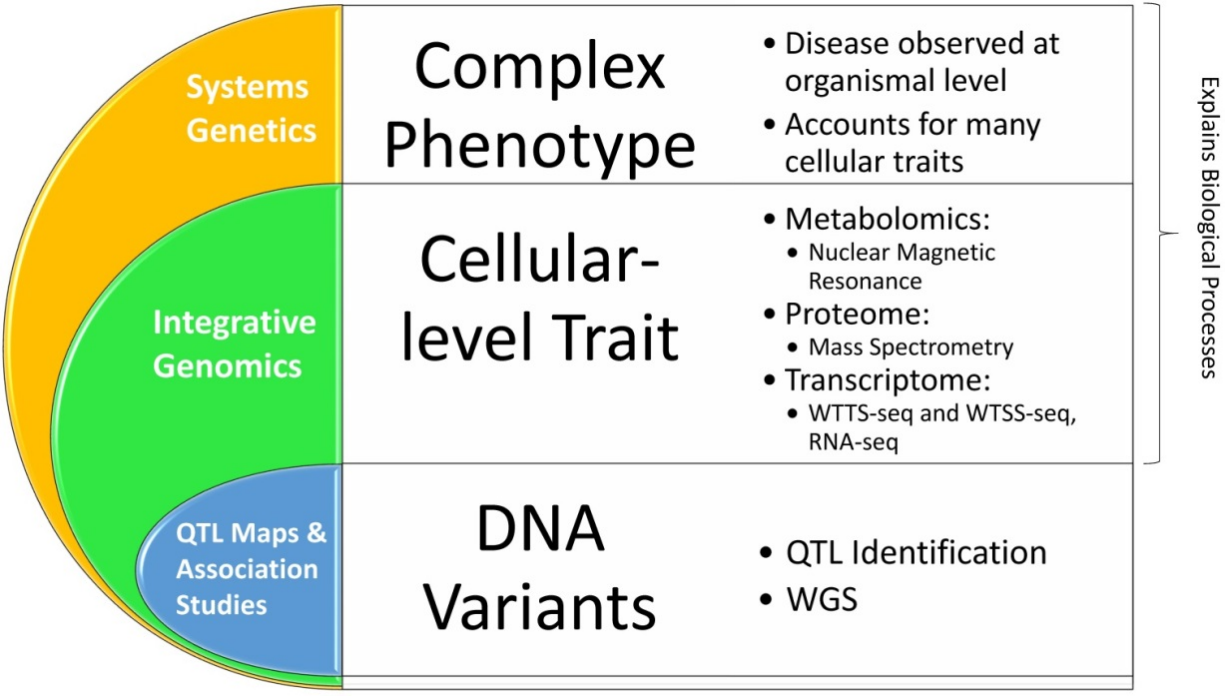
Advancements in rat genome-to-phenome research.

As discussed above, QTL mapping has been widely used to relate variants in the genome to variations in phenomes. However, this approach has two major limitations. First, the associations between genomes and phenomes established by QTL mapping cannot discern the molecular processes of cells and tissues that are disrupted, meaning they cannot be directly applied to development of new therapeutics 61. Second, a phenotype is usually controlled by multiple genes. As such, detection of associations between a single causal DNA variant and a complex phenotype can be misleading. For example, Baud and colleagues 29 observed that 44% of the analyzed QTLs in rats are due to the multiple gene effects, rather than single gene variants. Therefore, recognition of these insufficiencies coupled with the beginning of the 'omics' era resulted in the evolution of systems genetics, one of the best available methods for understanding the molecular pathways that link genomes to phenomes (**Figure 5**).

The first step towards systems genetics was extending QTL mapping to expression QTL (eQTL) analysis, which studies the associations between DNA loci and gene expression levels. Specifically, *trans*-eQTLs are eQTLs that are affected by genetic variants far away from the gene of interest, while causal genetic variants located close to or within the gene of interest are known as *cis*-eQTLs 27,47. Thus, eQTLs are more specific in describing how the genome is impacted to produce phenotypic changes based on gene expression. With respect to the rat, Atanur and colleagues 27 have identified genetic variations contained by cis-eQTLs affecting genes related to hypertension. The team observed that there was a significant enrichment (p < 10^-10^) of SNPs, large deletions, and INDELs in *cis*-eQTL gene regions as opposed to non-*cis*-eQTL gene regions as well as a significant enrichment (p < 2.2 × 10^-16^) of SNPs in the promoter region of *cis*-eQTLs compared to non-*cis*-eQTLs. Due to the success of eQTL mapping, QTL mapping has been further explored in DNA methylation (mQTLs), alternative splicing (sQTLs), chromatin accessibility (caQTLs), protein expression (pQTLs), cell metabolism (metaQTLs), ribosome occupancy (riboQTLs), histone (hQTLs), microRNA QTLs (miQTLs), and variance analysis (vQTL) 61-73.

Two important aspects are not addressed by these methods: 1) gene-gene interactions, gene-molecule interactions, and molecular pathways and 2) interactions between the genome and the environment. Thus, integrative genomics was piloted and soon became common due to technological advancements. With the goal of combining data from multiple different 'omics' focuses, integrative genomics used *in situ* analyses to discover the effects of certain genetic variants on one specific cellular trait such as RNA and protein expression levels (Figure 5) 71, 74-75. The large difference between integrative genomics and systems genetics is that systems genetics focuses on identifying the causal genetic variations behind complex disease phenotypes through computational modeling and observation of biological systems. While still focusing on cellular-level traits, systems genetics yields more comprehensive data because complex diseases usually affect multiple cellular traits that should be considered to fully understand the disease 71.

Thus, integrative genomics and systems genetics can account for gene × environment interactions as they document molecular reactions during cell or tissue analyses (**Figure 5**). They can determine gene regulation, co-expression and downstream gene regulation, epigenetic regulations, protein structure or expression changes, changes to mRNA and other RNAs that change their function, expression, stability or translation efficiency; and effects on co-regulated or downstream molecular pathways, depending on the research focuses and identified causal or contributing genetic variations/loci. To accomplish these approaches, molecular profiling is required and usually consists of gene expression analysis (RNA sequencing or RNA-seq), protein analysis (mass-spectrometry), and metabolite measure levels (nuclear magnetic resonance) 71, 76-79. Systems genetics then applies these data to known molecular interactions and pathways of cells and tissues, all of which describe genetic links to certain cellular traits that can be related to specific molecular pathways. For more information, Zheng and colleagues 72 created QTLbase, a database cataloguing cataloged QTLs based on their category, cell or tissue specificity, and known or speculated impacts on molecular pathways (see http://mulinlab.org/qtlbase).

#### Transcriptome diversity and dynamics

Several decades ago, genome-to-phenome events were simply described as “DNA makes RNA makes protein” by Francis Crick 80. However, many factors can dramatically complicate the processes. First, genome-to-phenome information transfer would not happen unless transcriptomes, proteomes and metabolomes are present to rapidly regulate epigenetic expression profiles in cells 81-83. Second, RNAs include not only coding (mRNAs, messenger RNAs), but also non-coding, such as tRNAs (transfer RNAs), rRNAs (ribosomal RNAs), snRNAs (small nuclear RNAs), snoRNAs (small nucleolar RNAs) and miRNAs (microRNAs) to execute diverse functions 84. Another category of RNAs is called long non-coding RNAs (lncRNAs) and their functions remain largely uncharacterized 85. Lastly, use of alternative promoters, isoforms and polyadenylation (polyA) sites dramatically amplify transcriptomes from a limited number of genes in genomes. In fact, timely use of transcript variants is essential to maintain differentiation and development and respond appropriately to environmental challenges, while the misuse of alternative transcripts often causes defects, diseases and disorders that affect nearly every system of the body 86.

Interestingly, a recent report revealed that transcriptome diversity is largely due to usage of alternative transcription start (ATS) and polyadenylation (APA) sites, rather than to alternative splicing 87. In humans, for example, roughly 200,000 ATS sites and 450,000 APA sites have been discovered 19,88. Two methods, whole transcriptome start and termini site sequencing (WTSS-seq and WTTS-seq) were specifically designed to profile either ATS or APA sites 89-91. Both methods are relatively simple and straightforward. In short, rRNA is either depleted or avoided, followed by enrichment of RNA targets, completion of first- and second-strand cDNA synthesis, and library sequencing. Overall, these processes make the protocols more accessible, easier to reproduce, and reduces the chances of technical error by reducing the instances in which errors or biases can occur.

To date, ATS sites in rats have been collected using the traditional CAGE (Cap analysis gene expression) method 19,92. The team used one universal RNA tissue sample, 6 aortic smooth muscle cell, 3 hepatocyte and 3 mesenchymal stem cell samples to perform the analysis. They obtained 28,497 'robust' and 92,031 'permissive' promoters for rat. Among 28,497 robust ATS sites, almost 98% were located in promoter (16,625 sites), intergenic regions (3,013), CpG islands (2,770), exons (1,937), introns (1,561), 5'UTRs (1,054), 3'UTRs (474) and TTSs (transcription termination sites, 403) 19. Derti and colleagues 20 discovered 52,128 known and 148,485 novel APA sites in rat brain and testis samples. Overall, relatively few studies on alternative transcripts have been performed in rats compared to humans and mice.

#### Using alternative transcriptomes to bridge the genome and phenome

Here we use our studies to show how alternative transcriptome analysis can provide evidence for phenome-related causal pathways in rats. The WTTS-seq method 89 for example, has at least five advantages over the traditional RNA-seq (RNA-sequencing) method. First, more than 99.9% of the reads produced by WTTS-seq are derived from polyA sites. Second, the diversity and dynamics of APA patterns across different time points/stages are clearly revealed by WTTS-seq, but are not detected by RNA-seq. Third, WTTS-seq authentically measures short transcript abundance, whereas RNA-seq reads are biased against both 5' and 3'-ends, making it difficult to accurately detect short transcripts 23. Fourth, when genes overlap, only WTTS-seq reads can be clearly assigned to corresponding genes because they are strand-specific. Lastly, intronic APAs can be revealed by WTTS-seq and evidenced by RNA-seq data. However, these sites are often missed if RNA-seq data are processed alone.

Using our WTTS-seq method, we recently examined how binge feeding a high fat diet (HFD) alters APA usage in the hypothalamus of male rats relative to control rodents fed a standard chow 21. In this study, 763 of the 89,022 APA sites revealed in the species were differentially expressed (DE-APA sites). Of these, 274 were down-, while 489 were up-regulated (*P*≤0.01), in rats fed the HFD compared to rats fed the control chow. Based on the differentially expressed genes (DEGs) assigned to these DE-APA sites, we observed that affected functional pathways were primarily related to neuron projection development and synapse organization. Phenotypically, HFD-exposed male rats showed characteristic hyperphagic feeding behavior by consuming significantly more calories than the controls in the early stage of the experiments, thus gaining an obese body weight relative to the controls in the later stage of the experiments. This implies that APA profiles can explain information flows from genome to phenome induced by an obesogenic environment.

The same WTTS-seq method was also used to detect how APA usages coordinate brain functions in response to cannabis exposure in rats 22. The study revealed that among 309 differentially expressed APA sites (*p* < 0.01), 243 sites were down-regulated while 66 were up-regulated in treated male rats in reference to the control animals. Pathway analysis showed that behavioral response and synaptic function are two main pathway clusters induced by marijuana exposure as compared to the air controls. Furthermore, the pathways in which APAs were differentially expressed between HFD and cannabis exposure were largely unshared, demonstrating the situational specificity of APA diversity and usage as well as the utility and informative value of transcriptome analysis. Therefore, alternative transcriptomes not only aid in understanding of complex gene functions, but also their reactions to the environment. As such, one of the most informative and common steps in associating genetic variations with disease or adverse phenotypes are transcriptome analysis (**Figure 5**). This cell-level phenotype is the first step in a cascade of molecular changes that result in disease phenotypes as opposed to their homeostatic counterparts.

## Conclusion and Prospects

Throughout this review we discussed the large WGS events and phenotype projects completed in the rat, resources where this information is available to the public, and the newest advancements in understanding of the genome-to-phenome events underlying complex traits. At the genome level, several ongoing sequencing projects will provide short- or long-reads that will minimize gaps and misplaced sequences, allowing the community to develop and fine-tune the rat pan-genome assembly. The significantly improved rat reference genome with its augmented structural accuracy and contiguity is evolving to full annotation with collection and characterization of genome-wide DNA variants for genetic analysis with health and disease phenotypes. Technically, proper sample sizes are required to represent the sequenced strain(s) and complete detection and characterization of polymorphic loci. In addition, the community needs to clearly define both INDELs and SVs in order to understand the published data and determine how to utilize them. For the phenotyping projects, both males and females need to be included as phenotypic disparities between sexes are common and significant. Characterization of the differences would enable researchers to advantageously exploit sex-specific characteristics, rather than treat them as confounding factors. No doubt, phenotyping both sexes is required to maintain the scientific premise and research vigor when human subjects and model organisms (including rats) are used in experiments. Sampling at different developmental and/or growth stages should be considered for phenotyping, because the time-serial datasets will help determine how multi-levels of intermediate phenotypes contribute to a higher-level phenotype. The comprehensive phenome datasets will also provide foundations for the rat research community to pursue phenome-wide association studies and thus unravel the pleiotropic effects that can link various phenotypes physiologically. No doubt, alternative transcriptome profiling is key to understanding the genome-phenome relationships because they transform the finite genome into the infinite phenome. The alternative transcriptome is situationally specific, and its dynamics are dependent on both the external and internal environment of the specifically analyzed cells. While systems genetics analyzes the transcriptome among other molecules (proteome and metabolome), the methodologies employed are sorely in need of improvements. However, methods such as WTSS-seq and WTTS-seq are currently being refined, improving both accuracy and sensitivity of detecting and mapping alternative transcripts. Thus, we recommend broad utilization of these types of methods in the future and that further methodological innovations of transcriptome analysis are considered.

## Supplementary Material

Supplementary table S1-S5.

## Figures and Tables

**Table 1:** Genome characteristics of human, rat and mouse

Parameter	Human	Rat	Mouse
Chromosome (pairs)	23	21	20
Size (Mb), first release	~2,900	~2,750	~2,600
Size (Mb), current release	3,088.3	2,782.03	2,725.52
Large segmental duplication	5-6%	3%	1-2%
GC% (median)	40.90	42.34	42.49
rRNA	29	5	62
tRNA	414	160	403
Other RNA	48,307	4,608	37,176
Gene	54,123	12,227	49,638
Pseudogene	16,186	2,570	10,441

**Table 2:** Characterization of genome-wide SNVs in rats

Genome location	Atanur et al., 2010	Guo et al., 2013	Hermsen et al., 2015
Intergenic region	2,251,679	1,150,467	6,509,332
Intron	1,064,314	837,376	2,991,180
Downstream	122,556	123,030	430,875
Upstream	143,546	119,219	427,613
3′ UTR	6,211	6,955	27,145
5′ UTR	2,571	1,061	4,357
Exon	31,781	20,963	74,151
Total	3,622,658	2,259,071	10,464,653
