# Supplementary material for: Genome-to-phenome research in rats: progress and perspectives
Source: Int J Biol Sci. 2021 Jan 1;17(1):119–33. doi: 10.7150/ijbs.51628 (PMC7757052; doi:10.7150/ijbs.51628)
Supplement: Supplementary file 1 — Supplementary table S1-S5. [file ijbsv17p0119s1.zip › ijbs_51628d2_2.docx]

Table S2. The U.S. Rat Phenome Collection

| **Cluster/No strains** | **Phenotypes** |
| --- | --- |
| Blood homeostasis trait/1 – 6 | blood hemoglobin level, blood pH, blood urea nitrogen level, mean corpuscular hemoglobin concentration, mean corpuscular hemoglobin level, partial pressure of blood carbon dioxide (Pco2), partial pressure of blood oxygen (Po2), plasma creatinine level, plasma glucose level, plasma insulin level, serum alanine aminotransferase activity level, serum albumin level, serum alkaline phosphatase activity level, serum aspartate aminotransferase activity level, serum bicarbonate level, serum calcium level, serum chloride level, serum creatinine level, serum globulin level, serum glucose level, serum phosphate level, serum potassium level, serum sodium level, serum total cholesterol level, serum total protein level, serum triglyceride level, serum urea nitrogen level and total serum bilirubin level |
| Body mass/44 | body weight |
| Body temperature trait/11 | body temperature |
| Circulatory system morphology trait/1 – 10 | artery tunica media width to artery inner diameter ratio, heart left ventricle weight to body weight ratio, heart left ventricle wet weight, heart right ventricle weight to left ventricle weight ratio, heart weight to body weight ratio and heart wet weight |
| Circulatory system physiology trait/4 – 13 | diastolic blood pressure, heart rate, mean arterial blood pressure and systolic blood pressure |
| Connective tissue morphology trait/1 – 2 | bone mineral content to body weight ratio, bone mineral density, epididymal fat pad weight, mesenteric fat pad weight, retroperitoneal fat pad weight and total volumetric bone mineral density |
| Gland morphology trait /6 – 7 | both adrenal glands weight to body weight ratio, both adrenal glands wet weight, both testes wet weight and both testes wet weight to body weight ratio |
| Grooming behavior trait/2 | duration of grooming in an experimental apparatus |
| Hemolymphoid system morphology trait/1 – 9 | blood eosinophil count, blood hemoglobin level, blood lymphocyte count, blood monocyte count, blood neutrophil count, hematocrit, mean corpuscular hemoglobin concentration, mean corpuscular hemoglobin level, mean corpuscular volume, red blood cell count, spleen weight to body weight ratio, spleen wet weight and total white blood cell count |
| Hepatobiliary system morphology trait/6 | liver weight as percentage of body weight and liver wet weight |
| Immune system morphology trait/1 – 6 | blood eosinophil count, blood lymphocyte count, blood monocyte count, blood neutrophil count, spleen weight to body weight ratio, spleen wet weight and total white blood cell count |
| Kinesthetic behavior trait/1 – 2 | amount of experiment time spent in a discrete space in an experimental apparatus, number of 20 x 20 cm floor squares crossed into, out of or within a discrete space in an experimental apparatus, number of entries into a discrete space in an experimental apparatus, number of rearing movements in an experimental apparatus and voluntary horizontal locomotion rate in an experimental apparatus |
| Molecule homeostasis trait/1 – 6 | blood hemoglobin level, mean corpuscular hemoglobin concentration, mean corpuscular hemoglobin level, plasma anion gap, serum alanine aminotransferase activity level, serum albumin level, serum alkaline phosphatase activity level, serum aspartate aminotransferase activity level, serum calcium level, serum chloride level, serum globulin level, serum phosphate level, serum potassium level, serum sodium level, serum total cholesterol level, serum total protein level, serum triglyceride level and urine protein excretion rate |
| Brain mass/6 | brain weight to body weight ratio and brain wet weight |
| Testis mass/6 | both testes wet weight and both testes wet weight to body weight ratio |
| lung mass/3 – 6 | lung dry weight to body weight ratio, lung weight to body weight ratio and lung wet weight |
| respiratory system physiology trait/9 | minute ventilation (VE), respiration rate and tidal volume |
| urinary system morphology trait/1 – 8 | artery tunica media width to artery inner diameter ratio, both kidneys wet weight, both kidneys wet weight as percentage of body weight, both kidneys wet weight to body weight ratio and urine protein excretion rate |
