# Supplementary material for: Genome-to-phenome research in rats: progress and perspectives
Source: Int J Biol Sci. 2021 Jan 1;17(1):119–33. doi: 10.7150/ijbs.51628 (PMC7757052; doi:10.7150/ijbs.51628)
Supplement: Supplementary file 1 — Supplementary table S1-S5. [file ijbsv17p0119s1.zip › ijbs_51628d2_3.docx]

Table S3. The Japanese Rat Phenome Collection

| **Cluster** | **Phenotypes** |
| --- | --- |
| Body Weight | 5 weeks (g), 6 weeks (g) and 10 weeks (g) |
| Home Cage Measurements | Body Position, Respiration, Tonic Involuntary Movement Cage, Vocalization and Palpebral Closure Cage |
| Hand-held Observations | Reactivity, Handling, Palpebral Closure Hand, Lacrimation, Salivation, Piloerection and Others |
| Open Field Activity | Rearings, Clonic Involuntary Movement, Tonic Involuntary Movement, Gait, Movements, Arousal, Occurrence of Stereotype, Abnormal Behavior, Defecations and Urinations |
| Stimulus Response | Approach Response, Touch Response, Eyelid Reflex, Pinna Reflex, Sound Response, Tail Pinch Response, Pupillary Reflex and Righting Reflex |
| Nervous and Muscle Measurements | Abdominal Tone, Limb Tone, Forelimb Grip Strength (N), Hindlimb Grip Strength (N) and Landing Foot Splay (mm) |
| Locomotor Activity | 0-10 min, 10-20 min, 20-30 min and 0-30 min |
| Passive Avoidance Test | Passive Avoidance Training (s) and Passive Avoidance Retention (s) |
| Blood Pressure and Body Temperature | Systolic Blood Pressure (mmHg), Heart Rate (1/min) and Body Temperature ( ‹C) |
| Blood Biochemistry | BW (g), GOT (IU/L), GPT (IU/L), ALP (IU/L), TP (g/dL), ALB (g/dL), A/G, Glu (mg/dL), T-CHO (mg/dL), HDL-C (mg/dL), LDL-C (mg/dL), TG (mg/dL), T-BIL (mg/dL), UN (mg/dL), CRE (mg/dL), IP (mg/dL), Ca (mg/dL), Plasma Na (mEq/L), Plasma K (mEq/L) and Plasma Cl (mEq/L) |
| Hematology | BW (g), RBC (x 10000/µL), Hb (g/dL), Ht (%), MCV (fL), MCH (pg), MCHC (g/dL), WBC (x100/µL), Platelets (x 10000/µL), PT (s), aPTT (s), WBC Bas. (%), WBC Eos. (%), WBC St. (%), WBC Seg. (%), WBC Lym. (%), WBC Mon. (%) and WBC Other (%) |
| Urine Parameter | Body Weight (g), Urine Volume (mL/6 Hrs), Volume/Body Weight (ml/100G/6Hrs), Na (mEq/L), Na/Body Weight (µEq/100G/6Hrs), K (mEq/L), K/Body Weight (µEq/100G/6Hrs), Cl (mEq/L) and Cl/Body Weight (µEq/100G/6Hrs) |
| Organ Weight | Brain (g), Brain (g%), Heart (g), Heart (g%), Lung (g), Lung (g%), Liver (g), Liver (g%), Kidneys (g), Kidneys (g%), Adrenals (mg), Adrenals (mg%), Spleen (g), Spleen (g%), Testes (g) ans Testes (g%) |
