# Supplementary material for: Genome-to-phenome research in rats: progress and perspectives
Source: Int J Biol Sci. 2021 Jan 1;17(1):119–33. doi: 10.7150/ijbs.51628 (PMC7757052; doi:10.7150/ijbs.51628)
Supplement: Supplementary file 1 — Supplementary table S1-S5. [file ijbsv17p0119s1.zip › ijbs_51628d2_4.docx]

**Table S4. Phenotypes collected for QTL mapping (Baud et al., 2013)**

| **Cluster/No animals** | **Phenotypes** |
| --- | --- |
| Anxiety/937 - 1375 | Distance 25' to 30', Distance first 5', Mean response latency, Number of avoidances, Number of crosses between trials, Time spent freezing, Latency to enter open section, Number of entries in open section, Stretched attend postures and Time spent in open section |
| Arterial elastic lamina ruptures/559 | Number of lesions in AA, Number of lesions in AA and IL, Number of lesions in IL, Score of lesions in AA, Score of lesions in AA and IL and Score of lesions in IL |
| Body weight/1226 | Body weight at day immunization |
| Bone morphology/1075-1174 | Axis length, Bone head width, Bone neck width, Distal femur cortical area, Distal femur cortical density, Distal femur total area, Distal femur total density, Distal femur trabecular area, Distal femur trabecular density, Femoral neck cortical area, Femoral neck cortical density, Femoral neck elongation, Femoral neck polar moment or inertia, Femoral neck stiffness, Femoral neck total area, Femoral neck total density, Femoral neck trabecular area, Femoral neck trabecular density, Femoral neck ultimate force, Femoral neck work to failure, Femur area, Femur elongation, Femur midshaft cortical area, Femur midshaft cortical density, Femur midshaft polar moment of inertia, Femur midshaft total area, Femur midshaft total density, Femur mineral content, Femur mineral density, Femur stiffness, Femur ultimate force, Femur work to failure, Length to femoral head, Length to trochanter, Lumbar area, Lumbar cortical area, Lumbar mineral content, Lumbar mineral density, Lumbar total area, Lumbar total density, Lumbar trabecular area, Lumbar trabecular density and Lumber cortical density |
| Cardiovascular function/1174-1386 | Blood pressure and Heart weight |
| Coat color/1407 | Is albino, Is dark brown, Is light brown and Is spotted |
| Glucose tolerance/930-938 | Area under glycemia curve, Area under glycemia curve over baseline, Glycemia 120' after injection, Glycemia 30' after injection, Glycemia 60' after injection and Glycemia before injection |
| Hematology/1293-1313 | Calculated mean cell haemoglobin concentration, Hematocrit, Hemoglobin, Hemoglobin distribution width, Lob-index, Mean corpuscular hemoglobin. Mean corpuscular volume, Mean peroxidase index, Mean platelet component, Mean platelet mass, Mean platelet volume, Measured mean cell hemoglobin concentration, Platelet clumps, Platelet component distribution width, Platelet count, Platelet distribution width, Plateletcrit, Proportion of basophils in WBC, Proportion of eosinophils in WBC, Proportion of large unstained cells in WBC, Proportion of lymphocytes in WBC, Proportion of monocytes in WBC, Proportion of neutrophils in WBC, Red blood cell count, Red blood cell distribution width and WBC in the Baso channel |
| Immunology/185-1248 | Absolute B cells, CD25+CD4+ cells, CD25+CD8+ cells, CD4+ T cells, CD8+ T cells and T cells; Expression of CD25 on C84+ cells, CD25 on CD4+ cells, CD28 on T cells, CD45 in CD4+ cells, CD45 in CD8+ cells, RT1A on granulocytes and RT1B on B cells; Proportion of B cells in in WBC, CD4-CD8- T cells, CD4+ cells expressing CD25, CD4+ cells in T cells, CD4+ cells with expressing CD45RC, CD4+ cells with high expression of CD25, CD4+ cells with high expression of CD45RC, CD4+ cells with low expression of CD45RC, CD4+ cells with not expressing CD45RC, CD4+CD8+ T cells, CD8+ cells expressing CD25, CD8+ cells in T cells, CD8+ cells with expressing of CD45RC, CD8+ cells with high expression of CD25, CD8+ cells with high expression of CD45RC, CD8+ cells with low expression of CD45RC, CD8+ cells with not expressing of CD45RC, T cells expressing RT1B and T cells in WBC; and Ratio of CD4+cells to CD8+ cells and T cells to B cells |
| Induced neuroinflammation/457-1405 | Balance disturbance, Cumulative scores, Died, Duration, First day with score>=1, Lowest weight, Maximum score, Symptoms for at least one day, Symptoms for at least two days, Weight loss compared to day 0 and Weight loss compared to day 9 |
| Renal agenesis/1407 | Renal agenesis |
| Serum biochemistry/1034-1365 | Alanine aminotransferase, Alkaline phosphatase, Aspartate aminotransferase, Basal glycemia, Calcium, Chloride, Creatinine, HDL, Iron, LDL, Potassium, Sodium, Total cholesterol, Triglycerides and Urea |
| Wound healing/1054 | Hole area |
