# Supplementary material for: Genome-to-phenome research in rats: progress and perspectives
Source: Int J Biol Sci. 2021 Jan 1;17(1):119–33. doi: 10.7150/ijbs.51628 (PMC7757052; doi:10.7150/ijbs.51628)
Supplement: Supplementary file 1 — Supplementary table S1-S5. [file ijbsv17p0119s1.zip › ijbs_51628d2_5.docx]

| \| ***Alcohol*** \| \| --- \| \| **Brain** \| \| 10 sodium channel genes^6^ \| \| 21 potassium channel genes^7^ \| \| 21 serotonin-related genes^7^ \| \| 27 potassium ion channel genes^6^ \| \| 4 glycine receptor genes^6^ \| \| 5 calcium ion channel genes^6^ \| \| 5 serotonin receptor genes^6^ \| \| 6 acetylcholine receptor genes^6^ \| \| ABCA1^9^ \| \| Agtr1a^1^ \| \| Axonal guidance pathways^7^ \| \| cAMP-mediated pathways^4^ \| \| Ces1^1^ \| \| Cholesterol synthesis pathway^6^ \| \| Corticotropin-releasing  hormone system^7^ \| \| CYP46A1^6^ \| \| Downstream CREB signaling  targets^7^ \| \| Drd2^8^ \| \| Fam111a^1^ \| \| FDFT1^9^ \| \| FDPS^9^ \| \| Gabra4^1^ \| \| Gene clusters related to Fos,  Nfkbia, and Srebf1^11^ \| \| Genes for 10 GABA-A receptors^7^ \| \| Glucocorticoid response  pathways^11^ \| \| Glutamate system genes^6^ \| \| Gng7^1^ \| \| Growth factor genes^7^ \| \| Hdac1,2,5^5^ \| \| HMGCoA^9^ \| \| Hmgcr^6^ \| \| Inflammatory response pathways^1^ \| \| Inflammatory response pathways^7^ \| \| Itpr1^1^ \| \| LTP/LTD^1^ \| \| Mx2^1^ \| \| Myelin producing genes^6^ \| \| Neuropeptide y system^7^ \| \| NMDA receptor genes^6^ \| \| Npy1r^6^ \| \| Oprk1^6^ \| \| Oprm1^6^ \| \| POMC^3^ \| \| Protein degradation pathways^11^ \| \| Protein kinase A signaling  pathways^4^ \| \| Renin-angiotensin signaling  pathway^1^ \| \| Slc5a7^1^ \| \| SREBF2^9^ \| \| Steroid hormone response  pathways^11^ \| \| Tac4^1^ \| \|  \| \| **Liver** \| \| Cholesterol synthesis pathway^2,9^ \| \| Cytoskeleton component  regulatory genes^2^ \| \| Egr-1^10^ \| \| Hdac2,4,5^5^ \| \| HMG-CoA reductase^2^ \| \| Phosphorylation of  ERK1,2 and RSK^10^ \| \| Lipid and ketone synthesis  pathways^2^ \| \| PAI-1^10^ \| \|  \| \| **Peripheral Blood** \| \| Hdac1-10^5^ \| \|  \| \| **Heart** \| \| Hdac1,7,8^5^ \| | \| ***Cannabis*** \| \| --- \| \| **Brain** \| \| Tyrosine hydroxylase activity^16^ \| \| Dopamine signaling pathways^12^ \| \| Drd2^14^ \| \| H3K14ac^18^ \| \| H3K9me2^18^ \| \| H3K9me3^18^ \| \| D1 and D2 dopaminergic receptors^16^ \| \| Mitochondrial function pathways^19^ \| \| Oxidative stress pathways^19^ \| \| Phosphorylation of Akt Thr 308, β-Catenin, GSK-3α/β, mTOR, p70S6 Kinase^17^ \| \| Slc6a3^12^ \| \| Synaptic function and transmission pathways^12^ \| \|  \| \| **Sperm** \| \| CB1^15^ \| \| c-MYC^15^ \| \| GLUT2^15^ \| \| Hippo signaling pathways^13^ \| \| Pancreatic \| \| PKB^15^ \| \| UCP2^15^ \| | \| ***Cocaine*** \| \| --- \| \| **Brain** \| \| 5-HT2cR^26^ \| \| arc^22^ \| \| ATF3^24^ \| \| ATF6^24^ \| \| AVP^25^ \| \| bdnf^22^ \| \| BIP^24^ \| \| c-fos^22^ \| \| CHOP^24^ \| \| DA D2R^26^ \| \| DNMT3a^28^ \| \| GADD34^24^ \| \| Genes of axon guidance molecules^27^ \| \| Genes of synaptic proteins^27^ \| \| HDAC2^28^ \| \| Oprk^21^ \| \| Oprm^21^ \| \| Pdyn^21^ \| \| Penk^21^ \| \| rBmal1^23^ \| \| rClock^23^ \| \| rCry^23^ \| \| rPer1^23^ \| \| rPer2^23^ \| \| rPer3^23^ \| \| Wnt signaling pathway^27^ \| \| XBP1^24^ \| \| zif268^20,22,26^ \| | \| ***Heroine*** \| \| --- \| \| **Brain** \| \| bdnf^30^ \| \| calb1^30^ \| \| dusp5,6^30^ \| \| egr1^30^ \| \| Fcrls^29^ \| \| npy^30^ \| \| rgs2^30^ \| \| Slc18a1^29^ \| | \| ***Methamphetamine*** \| \| --- \| \| **Brain** \| \| Alas1^36^ \| \| Aldolase A^37^ \| \| AP1^34^ \| \| Baiap2^37^ \| \| Basigin^37^ \| \| Bhlhe22^36^ \| \| CaM2^37^ \| \| Cell damage pathways^37^ \| \| Cellular metabolism pathways^37^ \| \| c-fos^33,34^ \| \| Cox-2^33^ \| \| CREB^34^ \| \| Egr3^31,34^ \| \| Erg1^34^ \| \| Erg2^33,34^ \| \| Fabp3^37^ \| \| fosb^31,34^ \| \| fra1,2^31,34^ \| \| GABAA receptor subunits α3, β1^35^ \| \| GABAB1 receptor^35^ \| \| GAT1,3^35^ \| \| Glycine receptor, subunit α2^37^ \| \| Importin 13^37^ \| \| Intercellular signaling pathways^37^ \| \| Intracellular signaling pathways^37^ \| \| junB^31,33,34^ \| \| Lipocalin 2^37^ \| \| Msx1^36^ \| \| Neurod1^36^ \| \| Nr4a1(Nur77)^34^ \| \| Nr4a2/nurr1^31,34,36^ \| \| Nr4a3(Nor-1)^33,34^ \| \| Olfactomedin-related ER localized^37^ \| \| Per2^32^ \| \| Preprotachykinin^34^ \| \| Rheb^37^ \| \| S100a8,9^36^ \| \| Sdfr1^37^ \| \| Sgk1 \| \| Syt10^33^ \| \| TRHR^37^ \| | \| ***Nicotine*** \| \| --- \| \| **Brain** \| \| BDNF^43^ \| \| Canonical pathways^38^ \| \| Chrna4,5,7^41^ \| \| Chrnb2^41^ \| \| Cym^42^ \| \| Drd1,2^41^ \| \| egr2^31^ \| \| fosb^31^ \| \| fra1,2^31^ \| \| Nervous system development pathways^38^ \| \| nr4a1/nurr77^31^ \| \| nr4a2/nurr1^31^ \| \| Perp^42^ \| \|  \| \| **Liver** \| \| GSK3β^40^ \| \| PEPCK genes^40^ \| \|  \| \| **Connective Tissue** \| \| Bone morphogenic protein-2^39^ \| \| Bone sialoprotein^39^ \| \| Core-binding factor α-1^39^ \| \| Osteopontin^39^ \| \| Type II collagen^39^ \| |
| --- | --- | --- | --- | --- | --- | --- | --- | --- | --- | --- | --- | --- | --- | --- | --- | --- | --- | --- | --- | --- | --- | --- | --- | --- | --- | --- | --- | --- | --- | --- | --- | --- | --- | --- | --- | --- | --- | --- | --- | --- | --- | --- | --- | --- | --- | --- | --- | --- | --- | --- | --- | --- | --- | --- | --- | --- | --- | --- | --- | --- | --- | --- | --- | --- | --- | --- | --- | --- | --- | --- | --- | --- | --- | --- | --- | --- | --- | --- | --- | --- | --- | --- | --- | --- | --- | --- | --- | --- | --- | --- | --- | --- | --- | --- | --- | --- | --- | --- | --- | --- | --- | --- | --- | --- | --- | --- | --- | --- | --- | --- | --- | --- | --- | --- | --- | --- | --- | --- | --- | --- | --- | --- | --- | --- | --- | --- | --- | --- | --- | --- | --- | --- | --- | --- | --- | --- | --- | --- | --- | --- | --- | --- | --- | --- | --- | --- | --- | --- | --- | --- | --- | --- | --- | --- | --- | --- | --- | --- | --- | --- | --- | --- | --- | --- | --- | --- | --- | --- | --- | --- | --- | --- | --- | --- | --- | --- | --- | --- | --- | --- | --- | --- | --- | --- | --- | --- | --- | --- | --- | --- | --- | --- | --- | --- | --- | --- | --- | --- | --- | --- | --- | --- | --- | --- | --- |

**Supplemental Table 5. Gene Expression Levels and Pathway Functions Altered by Substance Use Disorders in Rats.** Annotations of genes in the table correlate to the following citations: 1) Stankiewicz et al., 2015; 2) Klein et al., 2014; 3) Niikura et al., 2013; 4) McBride et al., 2014; 5) López‐Moreno et al., 2015; 6) McClintick et al., 2016; 7) McClintick et al., 2015; 8) Feltmann et al., 2018; 9) Alsebaaly et al., 2018; 10) Aroor et al., 2011; 11) McBride et al., 2013; 12) Brutman et al., 2019; 13) Murphy et al., 2018; 14) Di Nieri et al., 2011; 15) Levendal et al., 2012; 16) Fonseca et al., 1991; 17) Renard et al., 2017; 18) Prini et al., 2017; 19) Quinn et al., 2008; 20) Hollis et a., 2012; 21) Valenza et al., 2016; 22) Hearing et al., 2008; 23) Wang et al., 2019; 24) Pavlovsky et al., 2013; 25) Rodríguez-Borrero et al., 2010; 26) Besson et al., 2013; 27) Sillivan et al., 2011; 28) Zhou et al., 2014; 29) Imperio et al., 2016; 30) Kuntz-Melcavage et al., 2009; 31) Saint-Preux et al., 2013; 32) Natsubori et al., 2014; 33) Cadet et al., 2010; 34) McCoy et al., 2011; 35) Wearne et al., 2016; 36) dela Peña et al., 2013; 37) Ouchi et al., 2005; 38) Doura et al., 2010; 39) Yamano et al., 2010; 40) El.Golli et al., 2016; 41) Gozen et al., 2016; 42) Chen et al., 2014; 43) Castino et al., 2018.
